# Diabetic ketoacidosis as a complication of methanol poisoning; a case report

**DOI:** 10.1186/s12902-022-01037-z

**Published:** 2022-06-02

**Authors:** Azam Erfanifar, Mahsa Mahjani, Sara Salimpour, Nasim Zamani, Hossein Hassanian-Moghaddam

**Affiliations:** 1 Department of Internal Medicine, Loghman-Hakim Hospital, Shahid Beheshti University of Medical Sciences, Tehran, Iran; 2 Social Determinants of Health Research Center, Shahid Beheshti University of Medical Sciences, Tehran, Iran; 3 Department of Clinical Toxicology, School of Medicine, Loghman-Hakim Hospital, Shahid Beheshti University of Medical Sciences, Tehran, Iran

**Keywords:** Methanol, Poisoning, Diabetic ketoacidosis

## Abstract

**Introduction:**

Diabetic ketoacidosis (DKA) is a complication of diabetes presenting with high anion gap metabolic acidosis. Methanol poisoning, on the other hand, is a toxicology emergency which presents with the same feature. We present a case of methanol poisoning who presented with DKA.

**Case presentation:**

A 28-year-old male was referred to us with blurred vision and loss of consciousness three days after ingestion of 1.5 L of an unknown mixture of bootleg alcoholic beverage. He had history of insulin-dependent diabetes and had neglected his insulin shots on the day prior to hospital admission due to progressive loss of consciousness. Vital signs were normal and venous blood gas analysis showed severe metabolic acidosis and a methanol level of 10.2 mg/dL. After eight hours of hemodialysis, he remained unresponsive. Diabetic ketoacidosis was suspected due to positive urine ketone and blood sugar of 411 mg/dL. Insulin infusion was initiated which was followed by full awakening and extubation. He was discharged completely symptom-free after 4 weeks.

**Conclusions:**

Diabetic ketoacidosis and methanol poisoning can happen simultaneously in a diabetic patient. Given the analogous high anion gap metabolic acidosis, physicians should pay particular attention to examination of the diabetic patients. Meticulous evaluation for both conditions is highly recommended.

## Introduction

Methanol intoxication is a toxicologic emergency which occurs due to ingestion of methanol-containing alcoholic beverages in countries where alcohol ingestion is prohibited. Since treatments should be initiated immediately in case of high suspicion, it is important to recognize methanol poisoning promptly [1]. Any delay in initiation of treatment results in detrimental consequences including blindness and death. Symptoms include gastrointestinal and visual complications that can lead to critical involvement of the central nervous system and eventually to complete vision loss. Formation of formic acid is responsible for the high anion gap metabolic acidosis (HAGMA) [2].

Diabetic ketoacidosis (DKA) is one of the most debilitating complications of diabetes presenting with nausea, vomiting, dehydration, abdominal pain, acetone breath, and Kussmaul breathing pattern [3]. Diabetic ketoacidosis is also associated with HAGMA. It is imperative to differentiate DKA from other causes of metabolic acidosis [4]. We present a case of methanol poisoning presenting with DKA.

## Case presentation

A 28-year-old male presented to our emergency department with blurred vision and loss of consciousness three days after ingestion of 1.5 L of an unknown alcoholic beverage. He had a positive history of insulin-dependent diabetes mellitus and opium extract abuse. The patient’s companion declared that insulin shots had not been taken in the day prior to hospital admission. Physical examination revealed normal vital signs with a respiratory rate of 16 per minute. His first venous blood gas analysis (in the emergency department) showed severe metabolic acidosis with pH = 7.23, HCO_3_ = 7 mEq/L, BE = -19.1, pCO_2_ = 19 mmHg, and a methanol level of 10.2 mg/dL. Results of other lab tests are shown in Table 1.

**Table 1:** On-presentation Lab tests of the patient (Normal range)

Fasting blood sugar	411 mg/dL (< 100 mg/dL)
Serum ketone	+
Creatinine	3.1 mg/dL (0.59–1.04 mg/dL)
Urine drug testing	Opiates +
*Urine analysis:*	
pH	5.5
Color	Dark yellow
Appearance	Turbid
Blood	3 +
Ketone	1 +
Specific gravity	1.026
Bacteria	Many
Yeast cells	Many
Nitrite	Negative
WBCs	25–30
RBCs	Many
Urine culture	Candida
Serum lactate:	31 mmol/L (0.5–2.2 mmol/L)
*Bilirubin:*	
Total	1.8 mg/dL (0.1–1.2 mg/dL)
Direct	0.5 mg/dL (< 0.3 mg/dL)
*Serum Electrolytes:*	
Na	144 mEq/L (135–145 mEq/L)
K	4.5 mEq/L (3,6–5.2 mEq/L)
Ca	7 mg/dL (8.6–10.3 mg/dL)
P	2.8 mg/dL (3.4–4.5 mg/dL)
Mg	2.3 mg/dL (1.7–2.2 mg/dL)
*Liver function tests:*	
Ast	196 U/L (10–40 U/L)
Alt	257 U/L (7–56 U/L)
LDH	2125 IU/L (140–280 IU/L)
CPK	275 mg/dL (39–308 mg/dL)
Alkaline phosphatase	382 IU/L (44–137 IU/L)
*Complete blood count:*	
White blood cells	13.5 × 10^9^/L (4.5–11 × 10^9^/L)
Hemoglobulin	16.2 mg/dL (13.2–16.6 mg/dL)
Platelet	363 × 10^9^/L (150–450 × 10^9^/L)
ESR	4 mm/hr (0–22 mm/hr)
CRP	7.7 mg/dL (0.8–1 mg/dL)

**Fig. 1:**
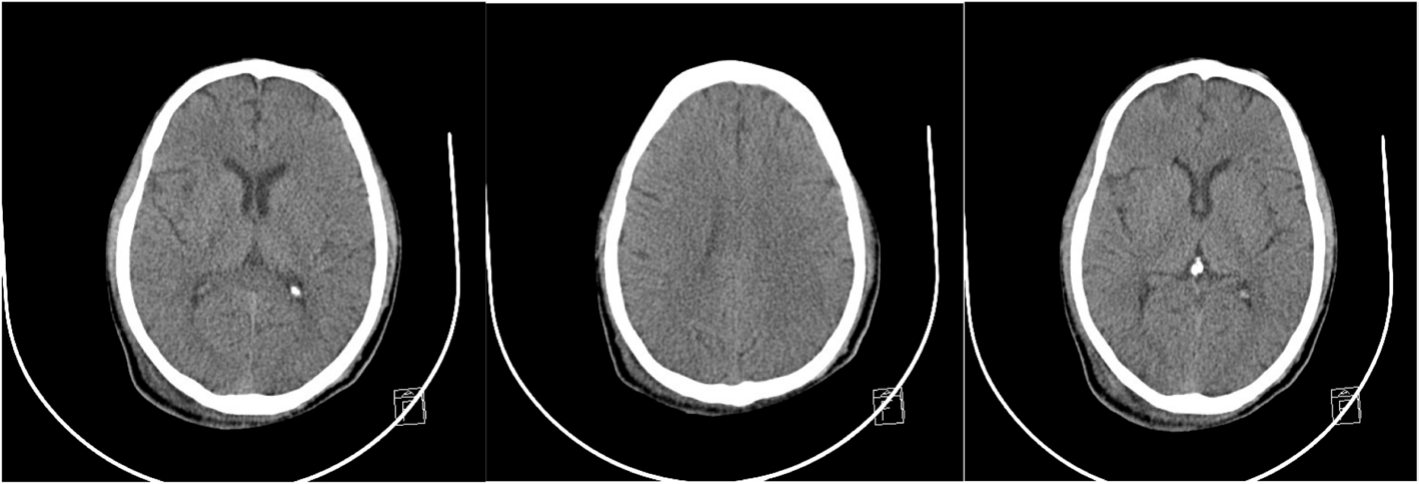
Brain computed tomography scan of the patient

**Fig. 2:**
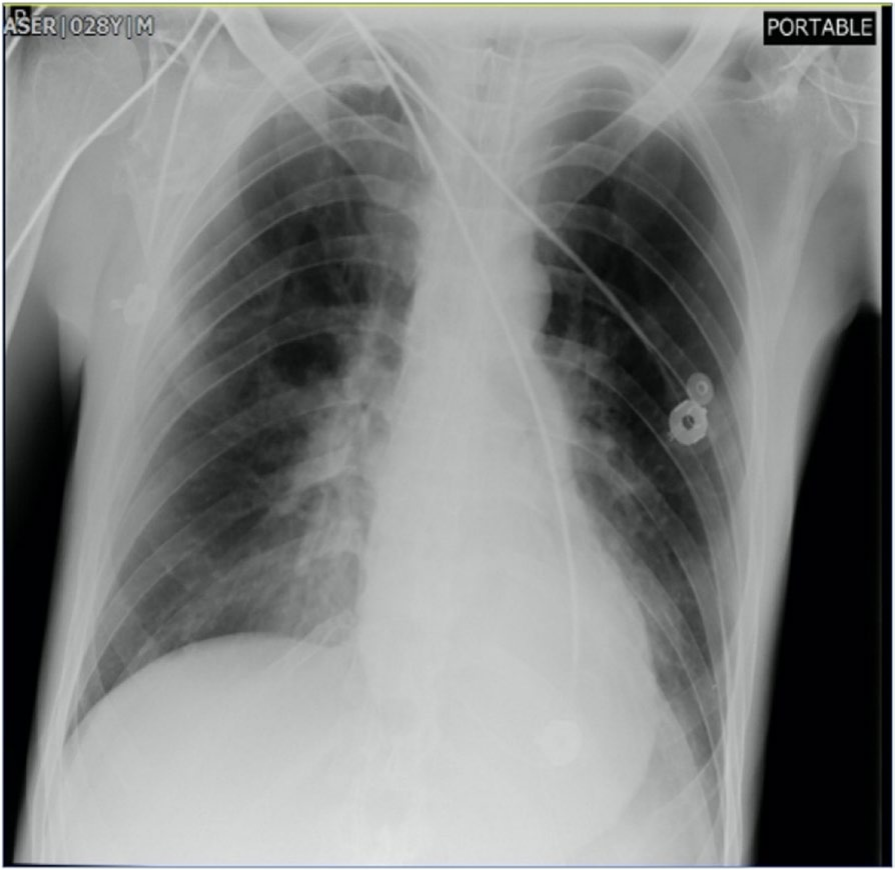
Chest X-ray of the patient

**Fig. 3:**
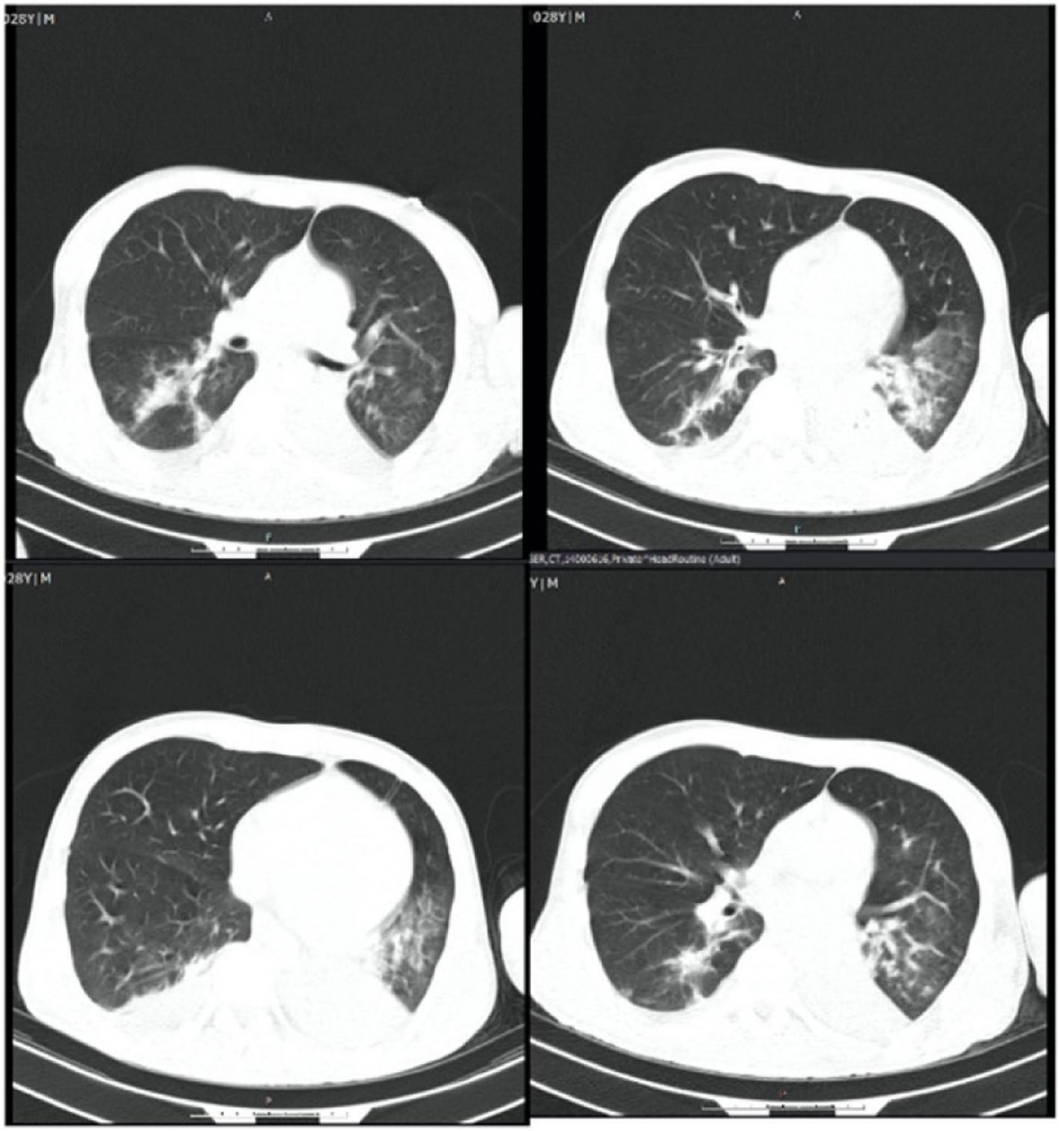
Lung computed tomography scan of the patient

**Fig. 4:**
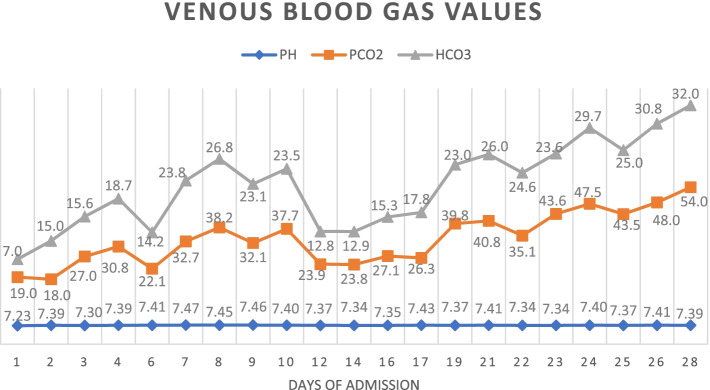
Daily blood gas analysis during hospital stays

**Fig. 5:**
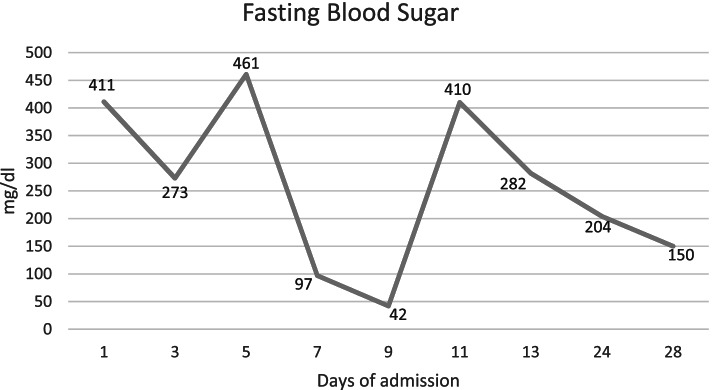
Fasting blood sugar changes during hospitalization

He was intubated and femoral catheterization was performed. Three vials of bicarbonate were intravenously administered. He underwent hemodialysis for eight hours but remained unresponsive. Serum/urine ketone and blood sugar were later returned to be positive and 411 mg/dL, respectively, indicating the possible presence of DKA. After initiation of insulin infusion, serum bicarbonate level started to rise. During admission, he had developed brain edema (Fig. 1). However, after appropriate treatment for DKA, the patient regained consciousness and was extubated. Since there were consolidations in his chest X-ray and lung computed tomography scan (Figs. 2 and 3), antibiotics and sepsis workup were ordered. He was initially treated by meropenem and vancomycin (for aspiration pneumonia); however, after consulting with the attending infectious disease specialist, he was started on levofloxacin. He was also receiving intravenous potassium, pantoprazole, heparin with prophylactic dose, and nebulized N-acetyl cysteine. Due to melena, endo-colonoscopy was performed but was reported to be normal. The other complication our patient experienced was deep venous thrombosis at the site of femoral catheter which mandated anticoagulation therapy with heparin drip. Diabetic ketoacidosis re-occurred twice more during the hospitalization. With the legitimate dose of methadone prescribed by psychiatrics, DKA completely resolved, and he was discharged home completely symptom-free after four weeks. Serial venous blood gas analysis, blood sugar and selected lab tests of the patient during hospital admission are shown in Figs. 4 and 5 and Table 2.

**Table 2:** Serial lab test results (during the first five days of ICU admission and thereafter; Hemodialysis was performed before admission to ICU)

	**Days of admission**						
	1st	2nd	3rd	4th	5th	10th	28th
**Creatinine (mg/dL)**	3.1	1.5	1.7	1.6	1.8	2	1.3
**K (mEq/L)**	4.5	4.4	3.5	3.0	3.9	3.6	4.5
**Na (mEq/L)**	130	137	140	141	146	142	138
**WBC (× 10**^**9**^** /L)**	13.5	8.6	7.6	7.6	8.9	9.3	7.2
**Hgb (mg/dL)**	16.2	13.3	12.1	12	11.3	11	11.2
**Platelet (× 10**^**9**^** /L)**	363	131	121	104	101	88	110
**Lactate (mg/dl)**	31	-	16	-	49	32	15

## Discussion and conclusions

Alcoholic beverages are prohibited in Islamic countries like Iran; therefore, methanol intoxication is rather prevalent in these countries [5]. Methanol is essentially found in various household and industrial chemicals [6, 7]. Methanol poisoning often happens due to its accidental ingestion and can be fatal if left untreated. This intoxication should be included in differential diagnoses of any altered mental status [8].

In low-income countries, toxicologists face obstacles including lack of laboratory tests to detect blood levels of methanol and its metabolites, making the diagnosis and management more complicated [9, 10]. Methanol is not toxic per se, while accumulation of its active metabolite, formic acid, plays a major role in development of metabolic acidosis [11]. In the late stages of intoxication, cellular damage is due to acidosis and histotoxic effect of formate which is accompanied by formation of the free radicals [12].

Diabetic ketoacidosis is a life-threatening acute condition [13] which more commonly occurs in type I diabetes [14]. If DKA overlaps with HAGMA due to any other reason, it may become challengingly difficult to diagnose DKA.

In this case scenario, methanol poisoning was accompanied by DKA in a diabetic patient. Such condition pronounces the hyperglycemic effect of methanol. On the other hand, DKA itself can be due to excessive exogenous acids like methanol. Additionally, stress-induced hyperglycemia is seen in critically ill and poisoned patients. In other words, increases in counterregulatory hormones as a result of acute stress caused by poisoning may contribute to hyperglycemia [15]. A study by Sanaei-Zadeh et al. [16] showed that there was significant correlation between blood glucose level and blood pH suggesting that acidosis could be associated with hyperglycemia. Acute methanol poisoning is a physical stress provoking pancreatic injury and can trigger diabetes in susceptible patients [15, 17].

It is important to highlight that opium addiction may also lead to severe hyperglycemia and an increase in insulin resistance [18]. Current literature suggests a possible association of opium withdrawal with aggravation of hyperglycemia, as well, although the relation was not well clarified [19, 20]. Therefore, screening for opium consumption in patients with recurrent DKA may be reasonable and beneficial.

Diabetic ketoacidosis and methanol poisoning can happen simultaneously in a diabetic patient. Given the analogous HAGMA, physicians should pay particular attention to examination of the susceptible individuals. Meticulous evaluation for both conditions is highly recommended. Accurate follow-ups should also be emphasized.

## Data Availability

Data are available on request. To have access to the data please contact: Email: hassanian@sbmu.ac.ir, Tel: + 982,155,409,534.

## Abbreviation

DKA: Diabetic ketoacidosis
